# Association between cognitive function and smartphone ownership among Japanese very old adults: a cross-sectional and longitudinal study

**DOI:** 10.1186/s12877-025-06561-w

**Published:** 2025-11-07

**Authors:** Naoki Takahiro, Yuko Oguma, Hisashi Urushihara, Azusa Hara, Takashi Sasaki, Yukiko Abe, Ryo Shikimoto, Yasumichi Arai

**Affiliations:** 1 Center for Supercentenarian Medical Research, Keio University School of Medicine, Tokyo, Japan; 2 Graduate School of Health Management, Sports Medicine Research Center, Keio University, Keio University, Tokyo, Japan; 3 Division of Drug Development and Regulatory Science, Faculty of Pharmacy, Keio University, Tokyo, Japan; 4 Faculty of Nursing and Medical Care, Keio University, Tokyo, Japan

**Keywords:** Cognitive function, MMSE score, Long-term care level, Digital literacy, Digital divide, Digital health

## Abstract

**Background:**

This research was conducted to investigate the association between cognitive function and smartphone ownership among very old adults, a rapidly growing age group. Additionally, we conducted a longitudinal investigation as a sub-analysis to determine whether owning a smartphone affects the level of long-term care certification as a proxy outcome for cognitive decline.

**Method:**

Data from a cross-sectional and longitudinal survey of the Kawasaki Aging and Wellbeing Project (KAWP) was used. Multivariate regression analysis was performed to examine the relationship between the Mini-Mental State Examination (MMSE) score and smartphone ownership. We also examined the association between smartphone ownership and the level of nursing care 1,278 days after the baseline.

**Results:**

Among 483 participants aged 85–90 years at baseline, 165 (46.9%) were male, and 131 (27.1%) owned a smartphone. In adjusted regression, smartphone ownership was associated with an MMSE score of ≥ 27 (odds ratio [OR]: 1.76, 95% confidence interval [CI]: 1.13–2.77), after controlling for potential confounders, including hypertension (OR: 1.65, 95% CI: 1.01–2.69). The results revealed no relationship between smartphone ownership and long-term care level.

**Discussion:**

Our results suggest that minimal cognitive decline in very old adults could make the ownership of a smartphone difficult.

**Supplementary Information:**

The online version contains supplementary material available at 10.1186/s12877-025-06561-w.

## Background

Revolutionary development in the field of digital communication technology in the past few decades has been observed worldwide. The technology has been integrated into various fields such as communication, shopping, and traveling, which has altered the daily lives of humans. This movement of digital transformation was pushed even more drastically with the coronavirus disease 2019 (COVID-19), as the overall Internet traffic increased by 30% from pre-COVID-19 to July 2020 [1], at which point several services began utilizing digital devices. During these transformations, aged people who did not grow up with these technologies are beginning to be left out of the digital world. The lack of digital literacy in aged people not only causes economic disparities, but studies conducted in China have revealed that digital literacy increases with increasing monthly household income per capita (RMB) [2]. In addition, health disparities among people with and without the knowledge of digital literacy widen as everyday information has to be gathered from the Internet as hospitals are using smartphones or tablets to take reservations or give out information to patients. Possessing eHealth literacy, which is defined as “the ability to seek, find, understand, and appraise health information from electronic sources and apply the knowledge gained to addressing or solving a health problem,” is positively related to health-related behavior suggesting the importance of the use of smartphones [3, 4]. While smartphone ownership—defined as possessing a smartphone regardless of usage frequency—and smartphone usage—defined as actually operating a smartphone for communication, information retrieval, or other purposes—are clearly distinct, ownership is commonly used as a proxy indicator for usage. However, smartphone shares among Japanese very old adults are low as only 17.3% of the population owned a smartphone in 2022 [5]. The same trend could be seen in other countries such as the US, which demonstrated that 17% of people ≥ 80 years owned a smartphone in 2016 [6]. Research in Australia and Slovenia showed similar results as only 3.3% of the population aged ≥ 65 years and 27.0% of the population aged ≥ 55 years owned a smartphone, respectively [7, 8]. In Japan, the population of very old is increasing at a high pace. Indeed, the overall proportion of Japanese aged ≥ 80 years was 10.5% in 2022, but is expected to increase to 15.6% by 2050 [9]. Identifying the factors that make it difficult for very old adults to have and use a smartphone, and improving the current disparities in digital literacy among the very old is now, more than ever, essential.

One major reason proposed for the low ownership of smartphones among very old adults is the decline in cognitive function. While most studies have investigated this link through patterns of smartphone use rather than ownership per se, these findings provide indirect evidence that cognitive ability plays a role in whether older adults are able to adopt and maintain smartphone use. A cross-sectional study conducted in China reported a strong correlation between smartphone use and better cognitive function as the proportion of cognitive impairment in “non-users of smartphone, dumb phone users, and smartphone users was 17.8%, 5.0%, and 1.4% respectively” [10]. Other research in China demonstrated that users of mobile devices had better cognitive scores and lower depression rates compared to non-users [11]. It has also been reported that in Singapore, focusing on frequency of use, people who use mobile phones “often” have better MMSE scores than those who “sometimes” or “never/rarely” use mobile phones [12]. The number of purposes a user gives to a smartphone is associated with cognitive function [13]. Moreover, research conducted in Japan stated that smartphone users had a higher processing speed than non-users [14], while research in France concluded that daily use of computers or other touchscreen devices leads to better cognitive function [15].

Most of these previous studies have focused on cognitive decline, often identified as mild cognitive impairment, defined as “cognitive decline greater than expected for an individual’s age and education level but that does not interfere notably with activities of daily life” [16]. However, there remains a lack of substantial data when focusing on participants aged ≥ 80 years, the demographic where cognitive decline is most commonly observed. Additionally, no research has specifically focused on this age group in Japan. Therefore, we hypothesized that minimal cognitive decline has an effect on the ownership of a smartphone in Japan. We selected a cut-off of MMSE ≥ 27 to define minimal cognitive decline based on a meta-analysis by Mitchell (2009) [17], which reviewed several studies assessing MMSE’s diagnostic accuracy for mild cognitive impairment (MCI). Notably, studies by Kalbe et al. and Xu et al. used a similar cut-off and demonstrated reasonable sensitivity and specificity for distinguishing MCI from healthy older adults and identifying those at risk of dementia. Considering our focus on detecting early cognitive changes specifically in very old Japanese adults, this threshold was chosen to balance sensitivity and specificity in this age group.

Moreover, for the very old, it is known from past studies that having more opportunities to gather information by themselves relates to delayed cognitive decline [18]. We additionally hypothesized that smartphone ownership may play a role in improving health by facilitating access to information and promoting engagement. Thus, to investigate the potential positive relationship between smartphone ownership and health outcomes, we analyzed long-term care insurance data as a proxy outcome.

## Methods

### Selection of participants

All the data used for the analysis were collected through the Kawasaki Aging and Wellbeing Project (KAWP) [19]. This is a longitudinal cohort study involving very old adults aged 85–89 years at enrollment. The participants were required to be residents of Kawasaki City and independent in the activity of daily living (ADL), which is indicated by having a level of care in the Japanese long-term care insurance system that is lighter than Support Level 1, and are able to visit to Kawasaki Municipal Hospital for baseline survey.

A total of 12,907 potential eligible participants were screened through basic registration of residents and the long-term care insurance database provided by Kawasaki City. Invitation letters were sent to 9,976 residents, 1,464 of whom responded positively, agreeing to take part in the research project. Finally, 1,026 participants were added to the KAWP between March 2017 and December 2018. Participants underwent a comprehensive baseline assessment, which evaluated their physical, mental, and cognitive functions, and psychosocial aspects. Subsequently, all participants were scheduled to be given a telephonic survey every 6 months, with the aim to check for the vitals, any incidents leading to disabilities, falls, and fractures, and hospitalization of the participants. The telephonic survey is planned to continue either until December 2024 or when the participants drop out beforehand.

**Fig. 1:**
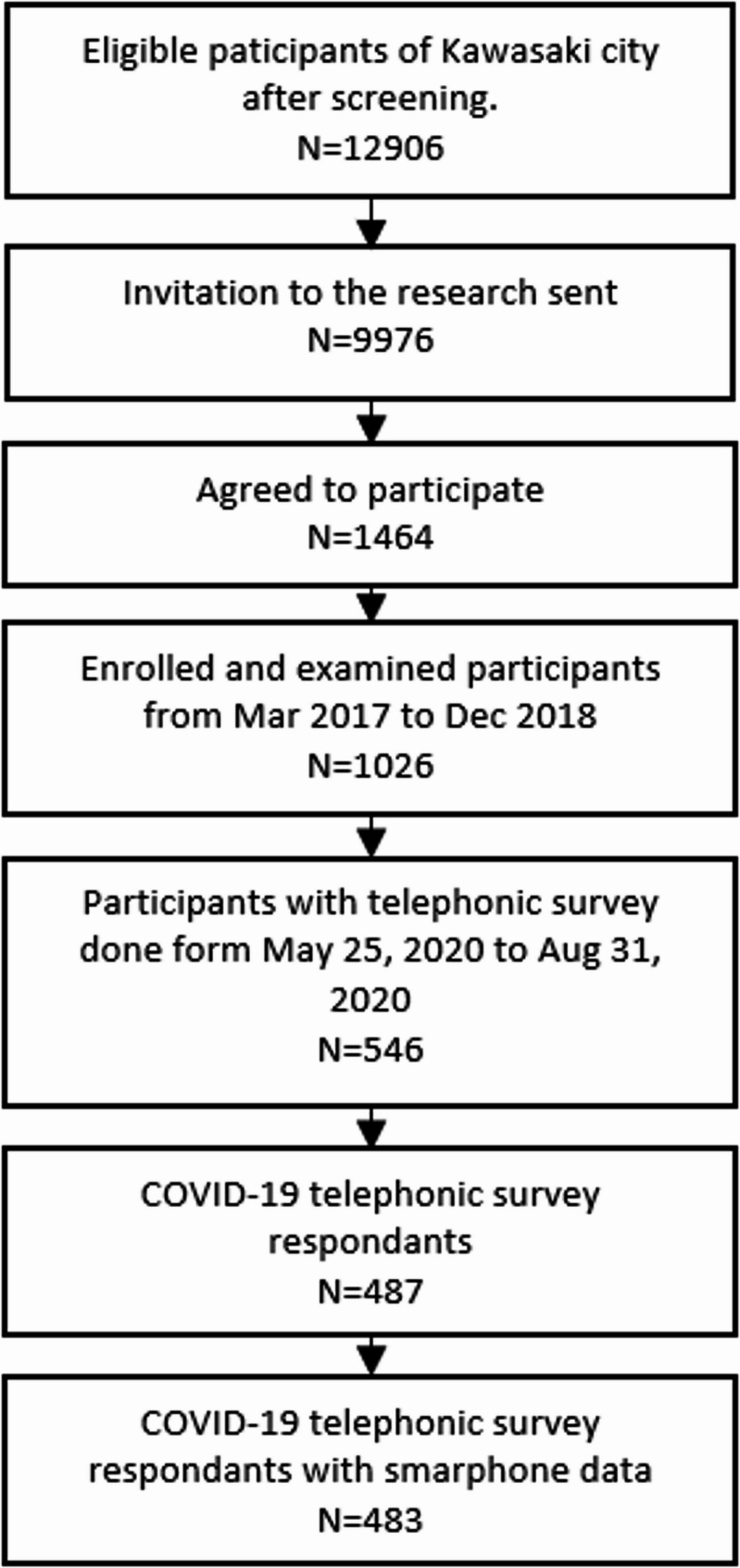
Flowchart of recruitment

In this study, we selected 546 participants who were scheduled for follow-up telephonic surveys between May 25, 2020, and August 2020. Among these participants, contact could not be established with 25 participants, and 34 declined, resulting in 487 participants. Moreover, four participants did not provide information regarding the ownership of smartphones, reducing the overall data used to 483 participants (Fig. 1) [20].]

The age and sex were acquired through official identification documents. Stroke, hypertension, diabetes, dyslipidemia, and hyperuricemia were measured through medical examinations conducted by trained physicians. MMSE and GDS were acquired through examinations conducted by trained psychologists. Lastly, other demographic characteristics, such as Education years, were provided through the self-reported questionnaire. All results above were collected between March 2017 and December 2018. Smartphone ownership was collected in a telephonic survey between May 25, 2020, and August 2020.

Written informed consent was collected from all KAWP participants, and the KAWP was approved by the ethics committee of the Keio University School of Medicine (ID: 20160297). The research protocol was adapted from the Tokyo Oldest Survey on Total Health and Japan Semi-supercentenarian Centenarian Medical Research, Keio University School of Medicine [21].

### Measurements

#### Smartphone ownership

Smartphone ownership was assessed through a telephonic interview. The participants were asked to respond with either “yes” or “no” to the questionnaire of whether they possessed their personal smartphone. Two trained interviewers conducted the telephonic interviews between May 25 and August 31, 2020, contacting participants through the contacts known in all cases for follow-up (stationary phones and cell phones were included). Interviews were conducted using a structured questionnaire on top of the 6-month recurring follow-up telephonic survey. Of all the questions, only the data for the ownership of personal smartphones was used in the analysis.

The assessment focused on ownership, rather than use. As smartphone ownership does not directly measure digital engagement, this is an important limitation of the analyses, which is also stated in the discussion. The binary ownership variable cannot capture the purpose or frequency of smartphone usage. Ownership alone may be an insufficient proxy for meaningful digital engagement, as emphasized here. However, it is known that smartphone ownership serves as a useful alternative indicator for initial adoption in the broader context of digital engagement [22].

### Baseline characteristics

#### Clinical and neuropsychological assessments

The KAWP baseline survey was conducted from March 2017 to December 2018, and participants visited one of three Kawasaki Municipal hospitals, including Kawasaki, Ida, and Tama. The questionnaire was distributed to the participants beforehand and included the following used in the analysis: age, sex, education years, living status (living alone or not), self-rated sensory function including vision and hearing, current jobs (yes or no), outgoing frequency in a week (more than two times), and care category on day 0. Financial hardship was measured on a scale ranging from 1 (struggling very much) to 5 (not struggling at all). Participants who answered “struggling very much” or “struggling somewhat” were classified as experiencing financial hardship [23]. Self-rated visual and hearing functions were measured on a scale ranging from 1 (very poor) to 5 (very good). Participants who answered “moderately poor” or “very poor” on their visual were classified as visually impaired, and participants who answered “need to be spoken to in loud voices” and “cannot hear at all” were classified as having a hearing impairment. All of the above groupings were made as they reflect meaningful financial and health difficulties. The presence of past medical history, including stroke, hypertension, diabetes, dyslipidemia, and hyperuricemia, was assessed through an interview conducted by a trained physician. The participant’s cognitive function was evaluated using the MMSE (0–30 points) [24], and the score in this study was cut off between < 27 and ≥ 27 based on previous research [16]. Depression was measured through the Geriatric Depression Scale (GDS) [25], with a cut-off of ≥ 5 indicating the presence of depressive symptoms.

The questionnaire was checked for consistency during the face-to-face interviews conducted at the survey sites. All data of a single participant used in the analysis were obtained on the same day.

#### Level of long-term care certificate

The level of long-term care was assessed based on the information provided by long-term care services operating under the long-term care insurance system in Japan. These services were categorized into two main groups: requiring support and long-term care. The requiring support category was further ranked into two levels, while the long-term care category was ranked into five levels. Individuals who did not use these services were categorized as self-dependent. Therefore, the participants’ ADL level was divided into the following nine categories: Self-dependent, requiring support levels 1–2, long-term care levels 1–5, and death. The long-term care certification dataset was initially collected at the baseline survey from March 2017 to December 2018. The endpoint was marked by the date participants were lost to follow-up, defined as moving out of Kanagawa prefecture, becoming a recipient of welfare benefits, or passing away. The primary outcome was the new certification of long-term care need (equivalent to care level 1 or higher). Data collected up to December 31, 2021, were used to determine whether participants had newly received long-term care certification [26]. Additional written informed consent to use the Kawasaki City database for research purposes was obtained from the participants.

### Statistical analysis

Characteristics of the baseline data are calculated as the mean and standard deviation. Participants with missing data were excluded from the analysis through listwise deletion. Categorical variables are expressed as numbers and proportions. Additionally, chi-square test and t-test were employed for categorical and continuous variables, respectively, to compare the participants’ characteristics by the ownership of smartphones. We conducted logistic regression analyses to assess the association between smartphone ownership and cognitive function, measured by the MMSE score. The analysis first calculated crude odds ratios (ORs) and 95% confidence intervals (CIs) for the association between smartphone ownership and MMSE score. Afterward, we considered potential confounding factors that might influence the results. As stated, a crude logistic regression model was first performed, which was followed by a multivariate regression model (model 1) that included MMSE (≥ 27) as the main explanatory variable, and was adjusted for sex and age. Subsequently, we made additional adjustments for depressive symptoms (GDS ≥ 5), education (≥ 13 years), visual impairment, and hearing impairment (model 2). Further adjustments included living status (living alone or not), current jobs (yes or no), and lifestyle-related diseases such as hypertension, diabetes mellitus, and dyslipidemia (model 3). Covariates were chosen as a relationship with cognitive function was reported in past studies [27]. These adjustments were made because smartphone ownership involves more complex instrumental ADL than just making a phone call, and these factors are possible influences on the ADL of very old adults. Lastly, because clinical diagnoses of dementia and depression were not collected, a sensitivity analysis was performed by operationally assuming an MMSE of less than 24 and a GDS of 10 or greater as a possible case of dementia. In Table 3, participants with “Requiring support level 1” or higher were selected as the outcome of the multivariate regression model. In this longitudinal study, we used multivariable logistic regression to examine the association between smartphone ownership and the incidence of long-term care certification (level 1 or higher) or death by December 2021. New certification for long-term care was used as a proxy indicator of cognitive decline, as cognitive impairment is a leading cause of long-term care need [28], while death was considered an outcome equivalent to the most severe level of care dependency or the termination of care. All results were considered statistically significant at a P-value of < 0.05. The MMSE license was obtained as MMSE-2 STANDARD BLUE FORM. SPSS Statistics ver. 29.0 software (Armonk, NY: IBM Corp.) was used for all analyses.

## Results

Of the 483 eligible participants for analysis, 49.5% (*n* = 239) were men. The mean age at baseline survey was 86.6 ± 1.37 years, ranging from 85 to 90 years. A total of 131 (27.1%) out of the 483 participants were using their own smartphones. Table 1 presents the participants’ characteristics based on smartphone ownership: participants without smartphones and participants with smartphones. The results showed no significant differences in sex, age, living arrangement (living with family or not), presence of children, frequency of going out, and financial hardship between smartphone owners and non-owners.

**Table 1:** Characteristics of the participants

	Does not have a Smartphone			Has a Smartphone			*P*
	(*n* = 352)			(*n* = 131)			
Age at first survey, (mean, std)	86.6		1.41	86.4		1.26	0.096
Sex							
Male, n (%)	165		46.9	74		56.5	
Female, n (%)	187		53.1	57		43.5	0.060
MMSE (mean, median, std)	25.8	27.0	2.81	26.6	27.0	2.66	0.004
MMSE ≥ 27, n (%)	149		42.6	76		58.5	0.002
GDS (mean, std)	3.43		2.95	2.58		2.33	0.001
GDS ≥ 5, n (%)	92		26.3	25		19.2	0.110
Education (mean, median, std)	10.8	10.0	3.07	12.3	12.0	3.36	< 0.001
Education ≥ 13 y, n (%)	89		25.3	52		39.7	0.002
Living status							
Alone, n (%)	98		27.8	32		24.4	
With someone, n (%)	254		72.2	99		75.6	0.452
Has children, n (%)	322		92.3	123		94.6	0.373
Frequency of leaving the Home in a week, n (%)							
None to 2	123		39.8	51		44.0	
More than 2	186		60.2	65		56.0	0.437
Currently has a job, n (%)	49		14.1	30		23.1	0.018
Financial hardship (Difficult), n (%)	271		78.8	106		82.8	0.331
Stroke, n (%)	32		9.2	16		12.2	0.322
Hypertension, n (%)	224		63.8	96		73.3	0.050
Diabetes Mellitus, n (%)	51		14.8	14		10.9	0.264
Dyslipidemia, n (%)	138		39.3	63		48.5	0.071
Hyperuricemia, n (%)	33		9.6	20		15.6	0.067
Visual impairment, n (%)	262		75.7	99		75.6	0.973
Hearing impairment, n (%)	20		5.8	15		11.5	0.033
Care category day 0, n (%)							
Independent	310		88.3	120		91.6	
Requiring support level 1	35		10	9		6.9	
Requiring support level 2	4		1.1	0		0	
Long-term care level 1	2		0.6	1		0.8	
Long-term care level 3	0		0	1		0.8	0.253
Care category day 1278, n (%)							
Independent	191		54.3	75		57.3	
Requiring support level 1	51		14.5	20		15.3	
Requiring support level 2	33		9.4	9		6.9	
Long-term care level 1	49		13.9	8		6.1	
Long-term care level 2	11		3.1	4		3.1	
Long-term care level 3	3		0.9	5		3.8	
Long-term care level 4	3		0.9	1		0.8	
Long-term care level 5	3		0.9	0		0	
Dead	8		2.3	9		6.9	0.023

All participants with missing values were excluded for the analysis

*Abbreviations:* *std* Standard deviation, *MMSE* Mini-Mental State Examination, *GDS* Geriatric Depression Scale

However, MMSE score (*p* = 0.004) and education years (*p* < 0.001) were positively associated with smartphone ownership. The difference in smartphone ownership rates between those scoring 27 or higher and those scoring below 27 was 12.6%. On categorical analyses smartphone owners had higher cognitive function (MMSE ≥ 27) (*p* = 0.002), fewer depressive symptoms (GDS ≥ 5) (*p* = 0.001), and a higher level of education (≥ 13 years) (*p* = 0.002), and showed a higher proportion of individuals with a job (*p* = 0.018) and hearing impairment (*p* = 0.033) (Table 1). The mean MMSE scores were 25.8 and 26.6, respectively, with 42.6% of no-smartphone participants scoring higher than 27, while 58.5% of smartphone owners scored higher than 27. The mean GDS score was 3.43 and 2.58, respectively, with 26.3% of no-smartphone participants scoring higher than 5, while 19.2% of smartphone owners scored higher than 5. The number (%) of participants with missing values were as follows: three (0.6%) in MMSE, three (0.6%) in GDS, four (0.8%) in Has children, 58 (12%) in Frequency of leaving the home in a week, five (1.0%) in Currently has a job, three (0.6%) in stroke, one (0.2%) in hypertension, ten (2.1%) in diabetes mellitus, two (0.4%) in dyslipidemia, 12 (2.5%) in hyperuricemia, six (1.2%) in visual impairment, five (1.0%) in hearing impairment, and one (0.2%) in Care category day 0 (Table 1).

Table 2, in which the purpose of the analysis was to determine the factors associated with smartphone ownership, shows the relationship between high cognitive function (MMSE ≥ 27)　and smartphone ownership as evaluated in logistic regression models. In both crude and all adjusted models, having a smartphone was positively associated with high cognitive function. In model 3, which adjusted for all potential confounding factors, having an MMSE score of 27 or more (OR: 1.76; 95% CI: 1.13–2.77), and hypertension (OR: 1.65; 95% CI: 1.01–2.69), were independently associated with having a smartphone (Table 2).

**Table 2:** Factors associated with smartphone ownership

	Crude		Model 1		Model 2		Model 3	
Characteristics	OR	95% CI	OR	95% CI	OR	95% CI	OR	95% CI
MMSE ≥ 27	1.90	1.26–2.86*	1.91	1.26–2.90*	1.65	1.07–2.54*	1.76	1.13–2.77*
Age	0.89	0.76–1.03	0.92	0.79–1.07	0.93	0.79–1.08	0.91	0.77–1.07
Sex	0.68	0.45–1.02	0.64	0.43–0.97*	0.73	0.48–1.13	0.71	0.44–1.14
GDS 5+	0.67	0.41–1.10			0.76	0.45–1.28	0.67	0.38–1.16
Education over 13 y	1.95	1.27–2.97*			1.55	0.98–2.46	1.59	0.98–2.59
Visual impairment	1.01	0.63–1.61			0.91	0.55–1.50	0.97	0.57–1.64
Hearing impairment	2.11	1.05–4.28*			1.92	0.92–3.99	1.87	0.86–4.06
Lives alone	0.84	0.53–1.33					0.83	0.49–1.41
Currently has job	1.83	1.10–3.04*					1.71	0.98–2.98
Financial hardship (Difficult)	1.30	0.77–2.20					1.03	0.57–1.86
Hypertension	1.56	1.00–2.42					1.65	1.01–2.69*
Diabetes mellitus	0.70	0.37–1.31					0.54	0.27–1.08
Dyslipidemia	1.45	0.97–2.18					1.47	0.92–2.35

*Abbreviations*: *OR* Odds ratio, *CI* Confidence interval, *MMSE* Mini-Mental State Examination, *GDS* Geriatric Depression Scale, *P < 0.05

Furthermore, to assess robustness while excluding the effects of dementia and depression, we conducted a sensitivity analysis involving sub-groups of participants, excluding those with MMSE scores less than 24 or GDS scores of 10 or more. In all models, having a smartphone was also positively associated with high cognitive function with an MMSE score of 27 or more (model 3, OR: 1.75; 95% CI: 1.05–2.92). Moreover, education years of 13 or more (OR: 1.90; 95% CI: 1.12–3.25), having a job (OR: 2.38; 95% CI: 1.29–4.38), and hypertension (OR: 2.00; 95% CI: 1.15–3.46) were all independently associated with having a smartphone in fully adjusted model 3 (Supplementary Table 1). With both Table 2 and Supplementary Table 1, having a smartphone constantly associated with the MMSE score, and education years of 13 or more, having a job, and hypertension marginally associated.

Table 3, in which the purpose of the analysis was to determine whether smartphone ownership affects the progress of long-term care level, presents the association between smartphone ownership and the utilization of long-term care services (defined as receiving certification for care level 1 or higher, or death) as of December 31, 2021, as assessed in the multiple logistic regression models. Participants who use service of long-term care level 1 or greater at baseline (0 days) were excluded from the analysis. The results showed no significant relationship between care level and having a smartphone (OR: 0.94; 95% CI: 0.60–1.47), as demonstrated in model 3 (Table 3).

**Table 3:** Factors associated with long-term care level

	Crude		Model 1		Model 2		Model 3	
Characteristics	OR	95% CI	OR	95% CI	OR	95% CI	OR	95% CI
Has smartphone	0.87	0.58–1.31	0.93	0.62–1.41	0.98	0.63–1.51	0.94	0.60–1.47
Age	1.18	1.03–1.35*	1.17	1.02–1.34*	1.18	1.03–1.35*	1.17	1.01–1.34*
Sex	1.51	1.05–2.16*	1.47	1.02–2.12*	1.77	1.19–2.63*	1.76	1.16–2.67*
MMSE 27 and above	0.68	0.47–0.97*			0.66	0.45–0.98*	0.65	0.43–0.97*
GDS 5+	1.81	1.19–2.75*			1.84	1.18–2.88*	1.70	1.08–2.69*
Education over 13 y	1.10	0.74–1.64			1.64	1.05–2.55*	1.68	1.06–2.66*
Visual impairment	1.00	0.66–1.53			1.06	0.68–1.67	1.11	0.70–1.77
Hearing impairment	1.45	0.73–2.86			1.71	0.83–3.54	1.68	0.79–3.57
Currently has a job	0.86	0.53–1.41					1.01	0.60–1.71
Hypertension	1.66	1.12–2.44*					1.52	0.99–2.33
Diabetes mellitus	1.57	0.92–2.67					1.45	0.82–2.57
Dyslipidemia	1.44	1.00–2.07					1.14	0.75–1.72

*Abbreviations*: *OR* Odds ratio, *CI* Confidence interval, *MMSE* Mini-Mental State Examination, *GDS* Geriatric Depression Scale, *P < 0.05

## Discussion

To the best of our knowledge, this is the first study to investigate the relationship between smartphone ownership and cognitive function in very old adults aged ≥ 85 years, while also examining ADL over more than 3 years. In the current study, we observed a correlation between smartphone ownership and MMSE score. The MMSE score cut-off was set at 27 or higher, suggesting that even a slight decline in cognitive function has a relationship with the ownership of smartphones in very old adults. The robustness of our results was demonstrated through the exclusion of participants with significant cognitive decline, which corresponds with dementia or depressive symptoms. Moreover, the ownership of smartphones did not have a relationship with ADL longitudinally. A noteworthy strength of our research lies in its dedicated focus on the previously understudied population of the very old, employing a robust cohort dataset to adjust for potential confounders of smartphone ownership, including psychosocial and physical factors. Previous research on the matter does not thoroughly focus on the very old age groups of ≥ 80 years, but does include a wide age range from 55 to 90 years [7, 8, 11], which does not focus on the very old precisely.

Our analysis involved 483 participants, with 27.1% of them owning a smartphone. In comparison, the research conducted in China included 21,732 participants with 14.3% of the participants owning a smartphone in 2017. It is difficult to compare the two as the demographics and the areas in which the data was collected are substantially different. However, in terms of focusing on the very old adults with high cognitive function, we believe that the high percentage of participants owning a smartphone supports the validity of our results.

Our main results were consistent with similar previous research [9, 10, 13]. The current research differed from previous research in terms of the focused age group; however, we believe that the similar result was due to the high level of cognitive function required when using a smartphone and the fact that the differences in age groups have less importance. In Table 2, a correlation between the MMSE score to education, having a job, and health complications could be observed in some models. The effect of sex becomes non-significant in Model 3, despite its significance in Model 1. Multicollinearity is one potential explanation; however, the Variance Inflation Factor (VIF) was below two for all variables, indicating that confounding by adjustment variables may be the primary cause. From past studies, it is known that mild to moderate decrements in cognitive functioning in non-demented persons are associated with diabetes, hypertension, obesity, and dyslipidemia [29], which indicates possible vascular cognitive decline with our result on hypertension and dyslipidemia. In other research, such as the one done in China [11], the correlation with hypertension or diabetes was not seen, suggesting the factors listed above have minimal relation to smartphone ownership in old people, but relate the very old. Although the ownership of smartphones associates with cognitive function, it could decrease physical activity and also add more stress to the very old [30]. Moreover, it may be able to be discussed that people experience physical and cognitive decline only from being old to very old, many experience an increase in comorbidities, fatigue, and a decline in ADL [31]. Furthermore, low social activity and contact are related to the increase in ones disabilities or physical function limitation [32]., suggesting that an intervention and education toward the very old is needed. Moreover, the lack of interaction leads to less information gathered for the very old, which may even influence the smartphone ownership.

Table 3 suggests that there is no relationship between smartphone ownership to subsequent long-term care level. However, age, sex, MMSE score, and GDS score all showed an association with smartphone ownership. Owning a smartphone is slightly shifted toward having a lower long-term care level from an OR of 0.94. Our findings suggest that smartphone ownership and long-term care level do not have a relationship due to the lack of physical activity due to the spread of COVID-19 [33], and also the difficulty that old people have in using smartphones in a way that increases their activity. Moreover, although it is known from past research that smartphones have a positive effect on self-rated health, it is also stated that age has a strong negative effect on self-rated health [34]. Our research focused on the very old; thus, it could be suggested that in the very old age group, the benefit of smartphones is minimal.

Intervention from a younger age using smartphones and other devices, resolving the study limitations, and increasing the dataset could lead to different outcomes.

As this study focused on a unique group of population, it has several limitations. First, the data collected for the research were limited, which weakens the reliability of the result. Second, there may be bias in the sample collected, as all of the participants were healthier than the same age group across Japan. Third, some of the criteria in the survey, such as hearing and vision, were self-rated, which would have reduced the accuracy. Furthermore, although the P-value for MMSE is significant (*p* = 0.004), the actual mean difference is modest (25.8 vs. 26.6) and the clinical significance is minimal. Consequently, the clinical significance may be constrained despite the statistical significance.

Moreover, although smartphone ownership is considered a valid alternative indicator of smartphone usage [22], the interview only asked whether the participant owned a smartphone or not, but did not go into detail about how often they use a smartphone or the purpose of the smartphone itself. In addition, there is a temporal discrepancy between the measurement of cognitive function (at baseline) and smartphone ownership, which was taken approximately 1.5 years later during the telephonic survey. However, in our population of very old adults (mean age ~ 87), smartphone ownership tends to be stable over time, particularly given the relatively low adoption and switching rates in this age group. Prior literature from Japan’s Ministry of Internal Affairs and Communication suggests that major changes in technology use are infrequent among the oldest-old, as the change in smartphone ownership rate from 2018 to 2020 was minimal, with the ownership rate being 7.8%, 11.0% and 11.0% respectively, in three years [35–37].

Finally, the causal relationship between smartphone ownership and MMSE score is unclear from a cross-sectional study. The results suggest that minimal cognitive decline could make the ownership of a smartphone difficult in very old adults. Additionally, we found no association between smartphone ownership and the subsequent long-term care level.

## Conclusion

These findings provide data for the understanding of the relationship between cognitive level and smartphone ownership and for the planning of how to increase the ownership rate of smartphones in aged people.

## Supplementary Information

Supplementary Material 1.

## Data Availability

The datasets analyzed in the current study will be made available upon reasonable request, subject to appropriate research arrangements and approval from the Ethics Committee of Keio University School of Medicine.

## Abbreviations

MMSE: Mini-mental state examination
KAWP: The Kawasaki Aging and Wellbeing Project
COVID-19: Coronavirus disease 2019
OR: Odds ratio
CI: Confidence interval
ADL: Activity of daily living
GDS: Geriatric depression scale
