# Supplementary material for: Association between cognitive function and smartphone ownership among Japanese very old adults: a cross-sectional and longitudinal study
Source: BMC Geriatr. 2025 Nov 7;25:866. doi: 10.1186/s12877-025-06561-w (PMC12595811; doi:10.1186/s12877-025-06561-w)
Supplement: Supplementary file 1 — Supplementary Material 1. [file 12877_2025_6561_MOESM1_ESM.pdf]

Supplementary Table 1 Factors associated with smartphone use, excluding MMSE <24 or GDS ≥10

	Crude		Model 1		Model 2		Model 3	
Characteristics	OR	95% CI	OR	95% CI	OR	95% CI	OR	95% CI
MMSE ≥27	1.83	1.15–2.89*	1.84	1.15–2.94*	1.64	1.01–2.67*	1.75	1.05-2.92*
Age	0.86	0.73–1.02	0.89	0.75–1.06	0.91	0.76–1.08	0.88	0.74-1.06
Sex	0.63	0.41–0.98*	0.61	0.39–0.96*	0.72	0.45–1.15	0.68	0.39–1.17
GDS 5+	0.90	0.52–1.58			1.05	0.59–1.89	1.02	0.54–1.91
Education over 13 y	2.17	1.37–3.43*			1.78	1.09–2.92*	1.90	1.12–3.25*
Visual impairment	1.09	0.65–1.83			0.99	0.58–1.72	0.99	0.55–1.79
Hearing impairment	2.11	1.00–4.43			1.89	0.87–4.11	1.79	0.78–4.11
Lives alone	0.89	0.53–1.48					1.00	0.55–1.84

Currently has job	2.49	1.42–4.35*	2.38	1.29–4.38*
Financial hardship (Difficult)	1.57	0.83-2.97	1.31	0.64-2.69
Hypertension	1.69	1.04–2.76*	2.00	1.15–3.46*
Diabetes mellitus	0.60	0.30–1.22	0.42	0.19–0.93*
Dyslipidemia	1.40	0.90–2.18	1.35	0.80–2.27

Abbreviations: OR: Odds ratio, CI: Confidence interval, MMSE: Mini-Mental State Examination, GDS: Geriatric Depression Scale, *P < 0.05.
